# A modality selective effect of functional laterality in pain detection sensitivity

**DOI:** 10.1038/s41598-021-85111-x

**Published:** 2021-03-25

**Authors:** Huijuan Zhang, Xuejing Lu, Yanzhi Bi, Li Hu

**Affiliations:** 1 CAS Key Laboratory of Mental Health, Institute of Psychology, Chinese Academy of Sciences, Beijing, 100101 China; 2 Department of Psychology, University of Chinese Academy of Sciences, Beijing, 100101 China

**Keywords:** Neuroscience, Psychology

## Abstract

The ability to detect environmental changes is essential to determine the appropriate reaction when facing potential threats. Both detection and reaction functions are critical to survival, and the superior performance of motor reaction for the dominant hand is well recognized in humans. However, it is not clear whether there exists laterality in sensitivity to detect external changes and whether the possible laterality is associated with sensory modality and stimulus intensity. Here, we tested whether the perceptual sensitivity and electrophysiological responses elicited by graded sensory stimuli (i.e., nociceptive somatosensory, non-nociceptive somatosensory, auditory, and visual) that were delivered on/near the left and right hands would be different for right-handed individuals. We observed that perceived intensities and most brain responses were significantly larger when nociceptive stimuli were delivered to the left side (i.e., the non-dominant hand) than to the right side (i.e., the dominant hand). No significant difference was observed between the two sides for other modalities. The higher sensitivity to detect nociceptive stimuli for the non-dominant hand would be important to provide a prompt reaction to noxious events, thus compensating for its worse motor performance. This laterality phenomenon should be considered when designing experiments for pain laboratory studies and evaluating regional sensory abnormalities for pain patients.

## Introduction

To avoid potential dangers, the vast majority of animals, including humans, use their senses to detect transient and sudden changes in their environments^1,2^. This function is critical to survival as the prompt detection of abrupt changes is crucial to determine the appropriate and immediate reaction to potential threats^3–6^. Regardless of the modality of sensory inputs, transient changes can elicit a series of brain responses, and the dominant part of the brain responses is a negative–positive biphasic vertex wave in the human electroencephalogram (EEG)^7,8^. The vertex wave has been proven to be highly associated with stimulus salience^9–11^, which represents the occurrence of stimuli contrasting relative to neighboring sensory inputs^12^. The saliency detection is considered as a key attentional mechanism that promotes survival by enabling individuals to focus their limited perceptual and cognitive resources on the most outstanding information^13–15^. In addition, the vertex wave, coupled with a complex modulation of the motor output^3^, also reflects neural processing important for the preparation and execution of defensive actions^16^.

In human biology, handedness, a preferential use of one hand over the other, is well recognized. Individuals normally prefer to use the dominant hand for a better motor performance. In contrast, the non-dominant hand is less preferred as it normally results in worse motor performance^17,18^. About 90% of the world’s population is right-handed^19^. Both detection and reaction functions are critical to survival. Even the superior performance of motor reaction for the dominant hand is well recognized, it is not clear whether the functional laterality due to handedness could also affect an individual’s sensitivity to detect transient changes. Specifically, it is still unclear whether the possible laterality in sensitivity to detect external changes is associated with sensory modality (i.e., nociceptive somatosensory, non-nociceptive somatosensory, auditory, and visual). Considering that pain is directly associated with real or potential bodily injury or tissue damage^20,21^, nociceptive somatosensory stimuli are believed to be more often followed by defensive actions (i.e., nocifensive behaviors) than sensory inputs of other modalities (i.e., non-nociceptive somatosensory, auditory, and visual stimuli). In addition to sensory modality, the saliency detection is highly associated with stimulus intensity, and strong nociceptive somatosensory stimuli are more often followed by defensive actions than weak nociceptive stimuli^22,23^. Therefore, the laterality of detection sensitivity to transient changes would be evident for strong nociceptive somatosensory stimuli, but not for weak nociceptive stimuli and sensory stimuli of other modalities.

To test these research hypotheses, we elucidated whether the perceptual sensitivity and electrophysiological responses elicited by transient sensory stimuli that were delivered on or near the left and right hands would be different for right-handed individuals. Specifically, graded sensory stimuli belonging to four different modalities (i.e., nociceptive somatosensory, non-nociceptive somatosensory, auditory, and visual) were delivered to a large number of healthy subjects (63 females and 37 males, 100 subjects in total) to assess whether the laterality phenomenon was associated with sensory modality and stimulus intensity.

## Results

### Subjective ratings of perceived intensities

Subjective ratings of perceived intensities evoked by stimuli of different sensory modalities (i.e., nociceptive somatosensory, non-nociceptive somatosensory, auditory, and visual) that were delivered to the left or right side are summarized in Table 1, and results of the two-way repeated-measures ANOVA are summarized in Table 2.

**Table 1:** Subjective ratings of perceived intensities, N1 and N2–P2 amplitudes, as well as ‘ERP’ and ‘α-ERD’ magnitudes elicited by nociceptive somatosensory (LEP), non-nociceptive somatosensory (SEP), auditory (AEP), and visual (VEP) stimuli that were delivered to the left and right sides (data are expressed as mean ± SEM).

Variables	Nociceptive (LEP)		Non-nociceptive somatosensory (SEP)		Auditory (AEP)		Visual (VEP)	
	Left	Right	Left	Right	Left	Right	Left	Right
Perception rating	5.20 ± 0.12	5.02 ± 0.13	3.75 ± 0.13	3.82 ± 0.13	4.76 ± 0.12	4.78 ± 0.12	3.95 ± 0.11	4.01 ± 0.11
N1 amplitude (μV)	− 4.76 ± 0.27	− 4.03 ± 0.24	− 9.82 ± 0.57	− 8.98 ± 0.55	–	–	–	–
N2–P2 amplitude (μV)	26.85 ± 1.07	24.81 ± 0.98	53.46 ± 1.70	52.36 ± 1.55	40.77 ± 1.38	41.39 ± 1.28	24.24 ± 0.83	24.37 ± 0.81
‘ERP’ magnitude (μV^2^/Hz)	11.42 ± 0.70	10.01 ± 0.66	15.84 ± 1.11	15.35 ± 1.11	14.69 ± 1.00	14.81 ± 0.96	4.13 ± 0.29	4.32 ± 0.31
‘α-ERD’ magnitude (μV^2^/Hz)	− 1.34 ± 0.21	− 1.00 ± 0.17	− 1.33 ± 0.22	− 1.26 ± 0.21	− 1.80 ± 0.24	− 2.13 ± 0.30	− 2.44 ± 0.37	− 2.35 ± 0.38

*LEP* laser-evoked potentials, *SEP* somatosensory-evoked potentials, *AEP* auditory-evoked potentials, *VEP* visual-evoked potentials.

**Table 2:** Results of two-way repeated-measures ANOVA with two within-subject factors (“sensory modality”: nociceptive somatosensory, non-nociceptive somatosensory, auditory, and visual; “stimulated side”: left and right sides).

Two-way ANOVA	Perception rating		N1 amplitude		N2–P2 amplitude		‘ERP’ magnitude		‘α-ERD’ magnitude	
	F	p	F	p	F	p	F	p	F	p
Modality	64.65	< 0.001	135.10	< 0.001	248.50	< 0.001	91.21	< 0.001	15.65	< 0.001
Stimulated side	0.02	0.889	7.05	0.010	1.25	0.226	1.53	0.220	0.14	0.710
Modality × stimulated side	4.35	0.008	0.08	0.772	3.43	0.022	3.40	0.021	4.11	0.012

**Figure 1:**
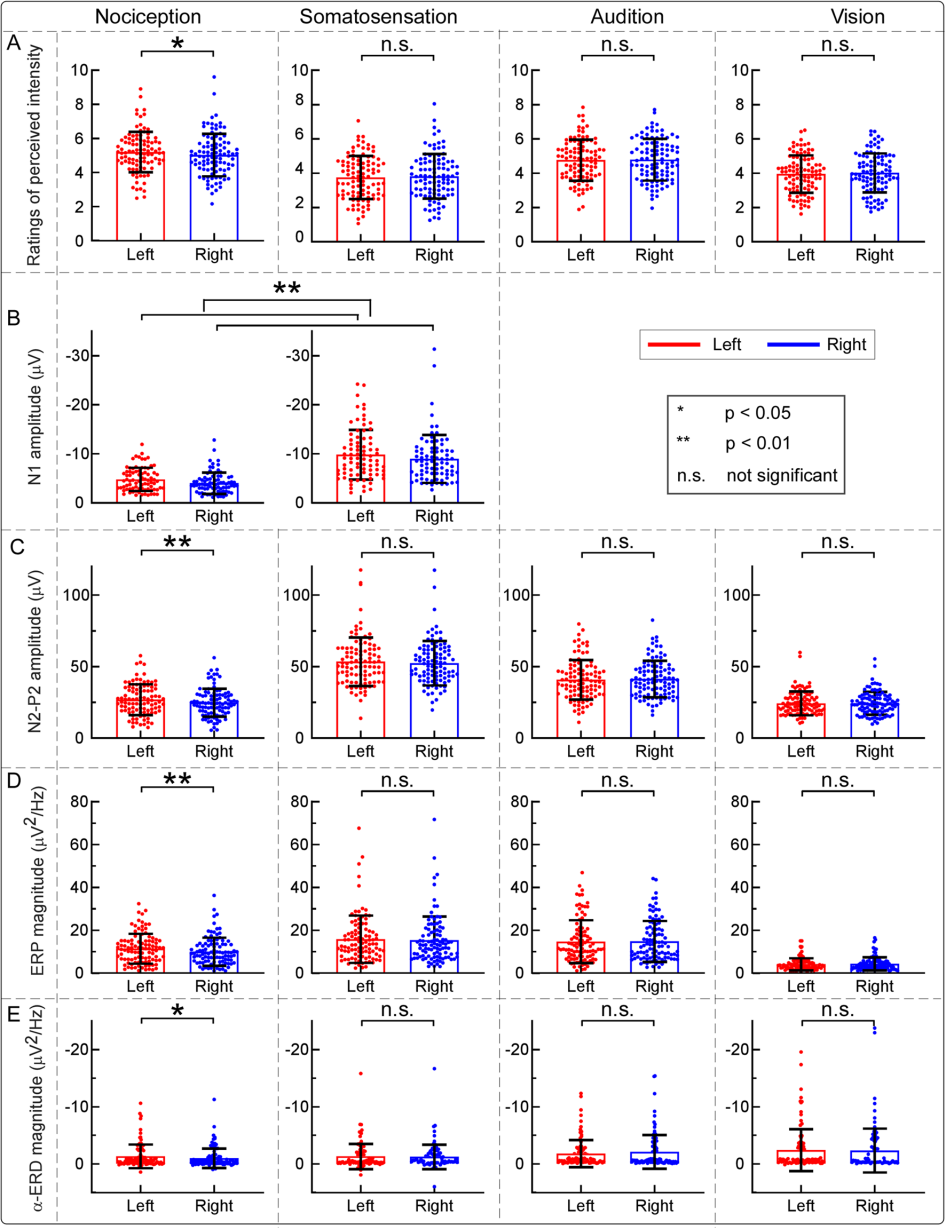
Effects of sensory modality and stimulated side on perceived intensities and electrophysiological responses elicited by transient stimuli. Perceived intensities, N1 amplitudes, N2–P2 amplitudes, ‘ERP’ magnitudes, and ‘α-ERD’ magnitudes elicited by nociceptive somatosensory, non-nociceptive somatosensory, auditory, and visual stimuli delivered to the left (red color) and right (blue color) sides are displayed from (**A**–**E**). Error bars represent SD (standard deviation) across subjects. Each dot represents the data from a single subject. Perceived intensities and almost all brain responses (N2–P2 amplitude, ‘ERP’ magnitude, and ‘α-ERD’ magnitude) elicited by nociceptive somatosensory stimuli that were delivered to the left side were significantly larger than those to the right side. In contrast, for non-nociceptive somatosensory, auditory, and visual stimuli, perceived intensities and brain responses were not significantly different between the two sides.

Two-way repeated-measures ANOVA revealed a significant main effect of “sensory modality” (*F*(2.60, 257.30) = 64.65, *p* < 0.001, *η*_*p*_^2^ = 0.40), and a significant interaction between the two factors (*F*(2.57, 245.82) = 4.35, *p* = 0.008, *η*_*p*_^2^ = 0.04). Post hoc paired-sample t-tests showed that the perceived intensity to nociceptive somatosensory stimuli that were delivered to the left side was significantly larger than to the right side (*p* = 0.010, Bonferroni correction, the same hereinafter). However, perceived intensities to non-nociceptive somatosensory, auditory, and visual stimuli were not significantly different between the two sides (*p* = 0.336, 0.644, and 0.232, respectively; Fig. 1A).

### Electrophysiological results in the time domain

**Figure 2:**
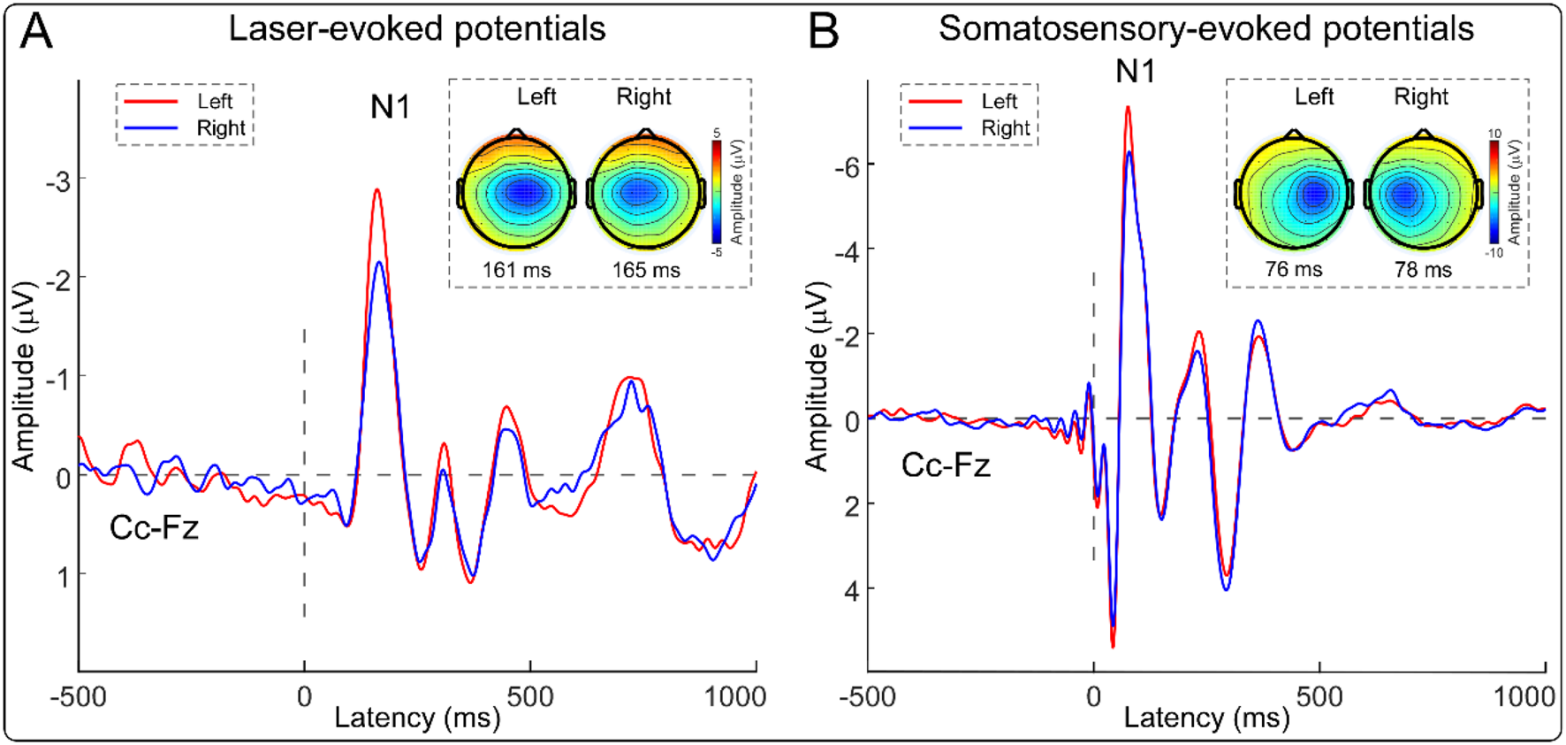
Group-level ERP waveforms and N1 scalp topographies elicited by nociceptive and non-nociceptive somatosensory stimuli. Group-level ERP waveforms (Cc-Fz) and N1 scalp topographies elicited by nociceptive somatosensory and non-nociceptive somatosensory stimuli that were delivered to the left (red lines) and right (blue lines) sides are respectively displayed in (**A,B**). The N1 amplitudes elicited by somatosensory stimuli delivered to the left side were significantly larger than to the right side.

**Figure 3:**
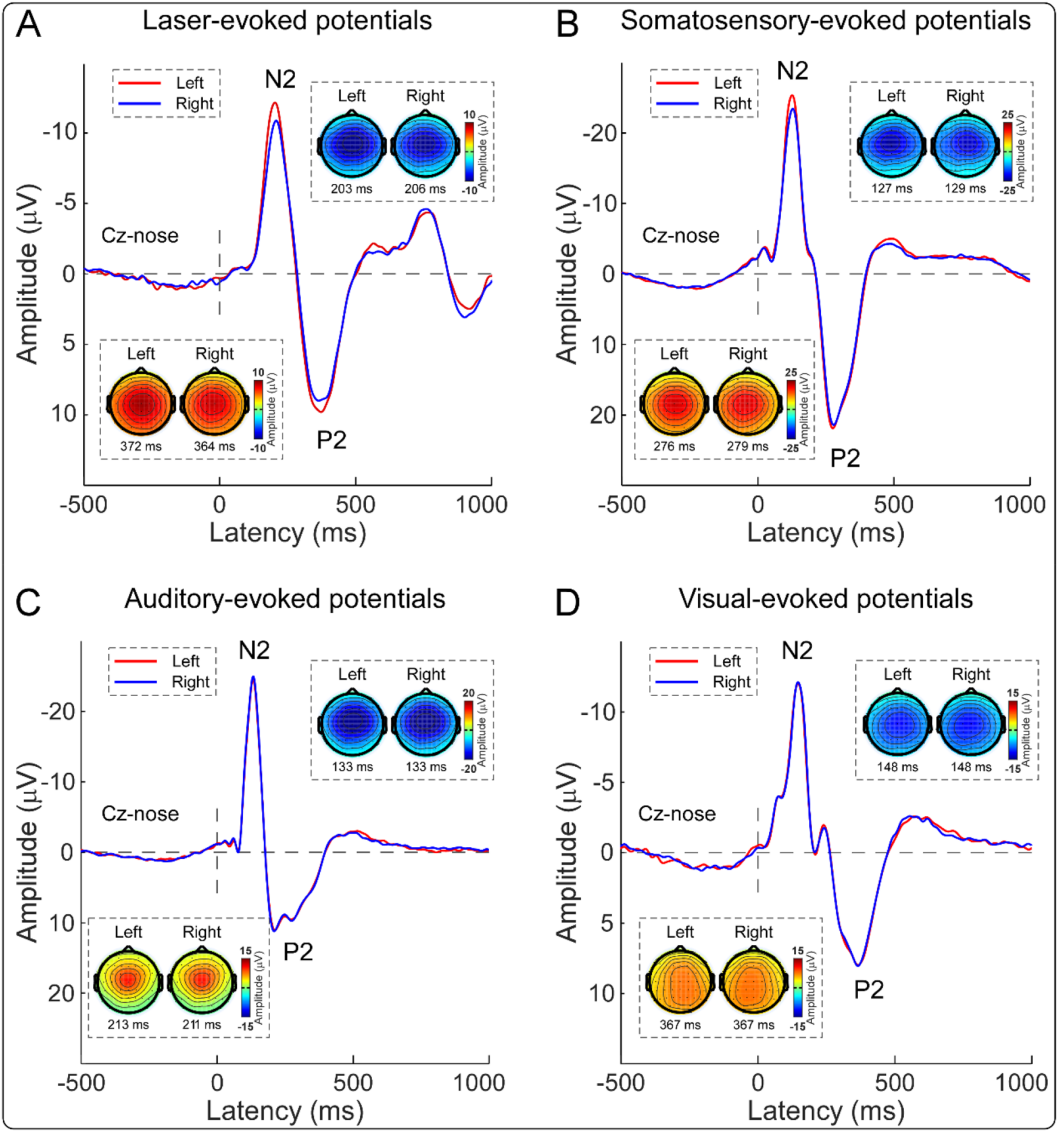
Group-level ERP waveforms and scalp topographies of N2–P2 vertex potentials elicited by nociceptive somatosensory, non-nociceptive somatosensory, auditory, and visual stimuli. Group-level ERP waveforms (Cz-nose) and scalp topographies of the N2 and P2 waves elicited by nociceptive somatosensory, non-nociceptive somatosensory, auditory, and visual stimuli delivered to the left (red lines) and right (blue lines) sides are displayed from (**A**–**D**). Whereas the N2–P2 amplitudes evoked by non-nociceptive somatosensory, auditory, and visual stimuli were not significantly different between left and right sides, the N2–P2 amplitude evoked by nociceptive somatosensory stimuli that were delivered to the left side was significantly larger than to the right side.

Group-level ERP waveforms and scalp topographies of N1, N2, and P2 waves are shown in Figs. 2 and 3. In line with previous studies^23–26^, scalp topographies of the N1 waves elicited by nociceptive somatosensory and non-nociceptive somatosensory stimuli were maximal at central electrodes contralateral to the stimulated side (Fig. 2). For all four sensory modalities, scalp topographies of the N2 wave were maximal at the vertex and extended bilaterally towards temporal regions, and scalp topographies of the P2 wave were more centrally distributed (Fig. 3). Peak amplitudes of the N1 wave and peak-to-peak amplitudes of the N2–P2 complex evoked by stimuli of different sensory modalities that were delivered to the left and right sides are summarized in Table 1, and the results of two-way repeated-measures ANOVA are summarized in Table 2.

For the N1 amplitudes elicited by nociceptive somatosensory and non-nociceptive somatosensory stimuli, there were significant main effects of “sensory modality” (*F*(1.00, 79.00) = 135.10, *p* < 0.001, *η*_*p*_^2^ = 0.63) and “stimulated side” (*F*(1.00, 79.00) = 7.05, *p* < 0.010, *η*_*p*_^2^ = 0.08). Specifically, the N1 amplitude elicited by non-nociceptive somatosensory stimuli was significantly larger than nociceptive somatosensory stimuli, and the N1 amplitude was significantly larger when somatosensory stimuli were delivered to the left side than to the right side (Fig. 1B).

For peak-to-peak amplitudes of the N2–P2 complex, two-way repeated-measures ANOVA revealed a significant main effect of “sensory modality” (*F*(2.57, 261.63) = 248.50, *p* < 0.001, *η*_*p*_^2^ = 0.72), and a significant interaction between the two factors (*F*(2.66, 263.44) = 3.43, *p* = 0.022, *η*_*p*_^2^ = 0.03). Post hoc paired-sample t-tests showed that the N2–P2 amplitude evoked by nociceptive somatosensory stimuli delivered to the left side was significantly larger than to the right side (*p* = 0.008; Figs. 1C, 3A), while the N2–P2 amplitudes evoked by non-nociceptive somatosensory, auditory, and visual stimuli were not significantly different between the two sides (*p* = 0.237, 0.455, and 0.816, respectively; Figs. 1C, 3B–D).

### Electrophysiological results in the time–frequency domain

**Figure 4:**
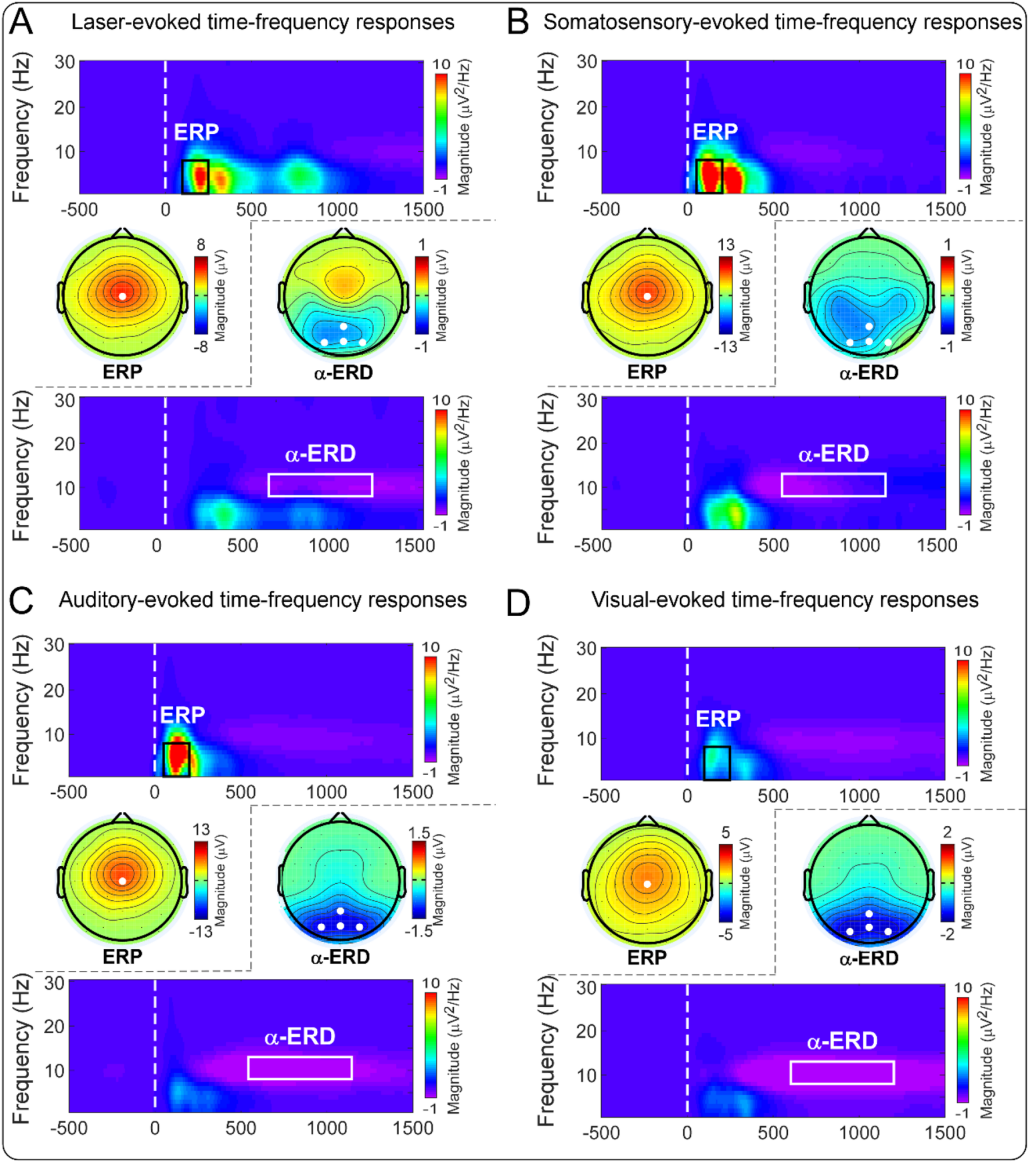
Group-level time–frequency distributions and scalp topographies of ‘ERP’ and ‘α-ERD’ responses elicited by nociceptive somatosensory, non-nociceptive somatosensory, auditory, and visual stimuli. Group-level TFDs and scalp topographies of the ‘ERP’ and ‘α-ERD’ responses elicited by nociceptive somatosensory, non-nociceptive somatosensory, auditory, and visual stimuli are displayed from (**A**–**D**). Regardless of sensory modality, transient stimuli elicited both phase-locked (‘ERP’: 100–250/50–200 ms, 1–8 Hz, central electrode) and non-phase-locked brain responses (‘α-ERD’: 600–1200/550–1150 ms, 8–13 Hz, parietal-occipital electrodes).

Group-level time–frequency distributions, together with the scalp topographies of the ‘ERP’ and ‘α-ERD’ responses elicited by stimuli belonging to four sensory modalities, are shown in Fig. 4. Consistent with previous studies^27–29^, all sensory stimuli elicited a large phase-locked response (‘ERP’, maximal at central midline electrodes) and a clear non-phase-locked response (‘α-ERD’, maximal at parietal-occipital electrodes, bilaterally). Please note that whereas the ‘α-ERD’ elicited by auditory and visual stimuli showed a negative maximum at occipital regions, the ‘α-ERD’ elicited by nociceptive and non-nociceptive somatosensory stimuli was likely to be contaminated by a distinct component that was distributed at bilateral sensorimotor areas^27^. The ‘ERP’ and ‘α-ERD’ magnitudes evoked by stimuli belonging to different sensory modalities delivered to the left or right side are summarized in Table 1, and the results of two-way repeated-measures ANOVA are summarized in Table 2.

For the ‘ERP’ magnitude, two-way repeated-measures ANOVA showed a significant main effect of “sensory modality” (*F*(2.48, 245.15) = 91.21, *p* < 0.001, *η*_*p*_^2^ = 0.48) and a significant interaction between the two factors (*F*(2.81, 278.21) = 3.40, *p* = 0.021, *η*_*p*_^2^ = 0.03). Post hoc paired-sample t-tests showed that the ‘ERP’ magnitude elicited by nociceptive somatosensory stimuli delivered to the left side was significantly larger than to the right side (*p* = 0.006; Figs. 1D, 4A), while the ‘ERP’ magnitudes elicited by non-nociceptive somatosensory, auditory, and visual stimuli were not significantly different between the two sides (*p* = 0.374, 0.832, and 0.294, respectively; Figs. 1D, 4B–D).

For the ‘α-ERD’ magnitude, two-way repeated-measures ANOVA showed a significant main effect of “sensory modality” (*F*(1.77, 175.63) = 15.65, *p* < 0.001, *η*_*p*_^2^ = 0.14) and a significant interaction between the two factors (*F*(2.44, 241.98) = 4.11, *p* = 0.012, *η*_*p*_^2^ = 0.04). Post hoc paired-sample t-tests showed that whereas the ‘α-ERD’ magnitude elicited by nociceptive stimuli delivered to the left side was significantly larger than to the right side (*p* = 0.041; Figs. 1E, 4A), the ‘α-ERD’ magnitudes elicited by non-nociceptive somatosensory, auditory, and visual stimuli were not significantly different between the two sides (*p* = 0.601, 0.061, and 0.608, respectively; Figs. 1E, 4B–D).

### The influence of stimulus intensity on the laterality of perceptual intensities elicited by sensory stimuli

Although no significant interaction among “sensory modality”, “stimulus side” and “stimulus intensity” (*F*(2.75, 271.82) = 1.94, *p* = 0.128, *η*_*p*_^2^ = 0.02) was found, three-way repeated-measures ANOVA showed significant main effects of “sensory modality” (*F*(2.60, 257.30) = 64.65, *p* < 0.001, *η*_*p*_^2^ = 0.40) and “stimulus intensity” (*F*(1.00, 99.00) = 1618.06, *p* < 0.001, *η*_*p*_^2^ = 0.94), and significant interactions between “sensory modality” and “stimulus intensity” (*F*(2.40, 237.58) = 78.30, *p* < 0.001, *η*_*p*_^2^ = 0.44), as well as between “sensory modality” and “stimulus side” (*F*(2.57, 254.82) = 4.35, *p* = 0.008, *η*_*p*_^2^ = 0.04). Post hoc paired-sample t-tests were conducted to break down the significant interaction between “sensory modality” and “stimulus side”, and the results showed that the perceived intensity to nociceptive somatosensory stimuli delivered to the left side was significantly larger than to the right side (*p* = 0.010), while such significant side effect was not found in non-nociceptive somatosensory, auditory, or visual modality (*p* = 0.336, 0.644, and 0.232, respectively; Fig. 1A).

## Discussion

In the present study, we investigated the possible functional laterality of detection sensitivity to transient changes on 100 right-handed subjects. One of the main findings of the present study is that the perceived intensities and almost all brain responses (the N2–P2 amplitude, the ‘ERP’ magnitude, and the ‘α-ERD’ magnitude) were consistently larger when nociceptive somatosensory stimuli were delivered to the left side than to the right side (Figs. 1, 2, 3, 4). This laterality was not observed for sensory stimuli of other modalities. This phenomenon should be considered when designing experiments for laboratory studies and evaluating regional sensory abnormalities in clinical practice.

### Laterality of sensitivity to detect transient changes of different sensory modalities

The effect of “stimulated side” on perceived intensities and brain responses (i.e., the N2–P2 amplitude, the ‘ERP’ magnitude, and the ‘α-ERD’ magnitude) to transient stimuli was quite different for different sensory modalities: they were significantly larger when nociceptive somatosensory stimuli were delivered to the left side than to the right side, while this laterality was not observed for transient stimuli of other sensory modalities (Figs. 1, 2, 3, 4). It should be noted that nociceptive somatosensory stimuli are more associated with defensive actions (i.e., nocifensive behaviors) than other stimuli, considering that pain is directly related to real or potential bodily injury or tissue damage^20,21^. The largest brain responses evoked by transient stimuli (i.e., the biphasic N2–P2 vertex potentials) are highly associated with stimulus salience^9,10^ and neural processing related to the preparation and execution of defensive actions^16^. The significantly larger intensity of subjective perception and larger amplitude of the vertex potentials elicited by nociceptive somatosensory stimuli delivered to the left side than to the right side would suggest that the non-dominant hand (i.e., left hand for right-handed subjects) was more sensitive in detecting transient noxious changes. The enhanced detection ability to nociceptive somatosensory stimuli is important to provide a prompt reaction to the noxious event, thus compensating for the worse motor performance of the non-dominant hand. Importantly, the dominant hand has superior motor performance over the non-dominant hand^17,18^, which has been previously used to explain why the dominant side can withstand more pain^17,30,31^. The reduced sensitivity to nociceptive somatosensory stimuli of the dominant hand would also be associated with the long-term adaptation during the evolution process, as the dominant hand is more frequently used to detect the threats in daily activities^32^.

However, the effect of “stimulated side” on perceived intensities and brain responses was not observed for transient stimuli of other sensory modalities. This negative observation could be associated with the fact that transient stimuli of other sensory modalities (i.e., non-nociceptive somatosensory, auditory, and visual) are of less association with defensive behaviors than nociceptive stimuli^20,21^. In addition, the negative “stimulated side” effect for non-nociceptive sensory modalities could also be associated with the factor that the stimuli were delivered near the body and not lateralized enough. Indeed, to ensure that brain responses are comparable across different sensory modalities, auditory and visual stimuli were delivered near left or right hand in our study. The distance between the source of stimulation and the head, as well as the distance between both hands were fixed (approximately 60 cm). This experimental design is different from previous studies in which sensory stimuli were delivered to one ear/eye^33^. Such difference would result in different outcomes between our study and previous studies^34,35^.

Contrary to our hypothesis, no significant difference in the laterality effect was found between transient nociceptive somatosensory stimuli with low-intensity and those with high-intensity. It should be noted that such negative outcome, however, does not guarantee that the laterality in response to nociceptive somatosensory stimuli cannot be affected by stimulus intensity, as it is possible that the manipulation of the stimulus intensity gradient in the present study failed to distinguish their biological significance.

### Possible mechanisms underlying the laterality of sensitivity to detect transient nociceptive stimuli

The laterality of sensitivity to detect transient nociceptive stimuli is supported by many previous studies, which showed that healthy subjects had higher pain sensitivity, e.g., decreased pain threshold^17,31,36–38^ and pain tolerance^30,36,37,39^ as well as increased subjective ratings of pain intensity^40,41^, when different types of noxious stimuli were delivered to the non-dominant hand, including pressure^17,31,38^, electrical^37^, cold^30,39^, and heat^36,40,41^ stimuli. In addition, clinical observations also showed the laterality phenomenon, represented as stronger pain and a higher rate of pain occurrence at the non-dominant than the dominant body sites, in patients with pain conditions^42,43^.

Intuitively, this laterality phenomenon to nociceptive stimuli can be interpreted by two possible mechanisms. First, at the peripheral level, use-dependent changes of the thermal and mechanical properties of the skin and the underlying tissues of the dominant hand (e.g., the skin fold and the sensitivity of nociceptors) could be partly responsible for our observation^41^. Indeed, we observed that the N1 wave, largely generated from the primary somatosensory cortex (S1) contralateral to the stimulated side^23,25^, showed larger amplitude when somatosensory stimuli were delivered to the non-dominant hand than to the dominant one (Tables 1, 2, Figs. 1B, 2). Since the N1 wave represents somatosensory specific activities maximally reflecting the incoming sensory inputs^44^, it is likely that compared to the dominant hand, the information raised by the stimulation of the non-dominant hand was more effectively transmitted in the peripheral pathways in the somatosensory systems. Please note that the peripheral mechanism was challenged by some studies, which showed the lack of laterality effect in left-handed subjects^17,30,31^. However, the reasoning underlying this challenge may not hold, since both right-handed and left-handed subjects are living in a predominantly right-hand society^17,32,40^, and use-dependent changes of the thermal and mechanical properties of the skin and the underlying tissues of the dominant hand would not be equal for both types of subjects.

Second, at the central level, it has been interpreted that the laterality of sensitivity to detect transient nociceptive stimuli would be associated with the greater involvement of the right brain hemisphere in processing negative emotions and aversive states than the left brain hemisphere^30,45^. This interpretation suggested that the greater sensitivity of the non-dominant (left) hand to nociceptive somatosensory stimuli would reflect cerebral laterality in the emotional and aversive aspects of pain^17,31,36,38,40^. In addition, regardless of the stimulated side of the nociceptive somatosensory stimuli, predominant right hemisphere activation was also observed in some other brain regions, e.g., the thalamus and secondary somatosensory cortex^46^, which are mainly responsible for the sensory/discriminative aspects of pain^41,47^. Both central factors (emotional/aversive and sensory/discriminative aspects of pain) would subserve the neural basis for the unique role of the right brain hemisphere in pain processing, which results in greater perceived intensities and larger brain responses (the N2–P2 amplitude, the ‘ERP’ magnitude, and the ‘α-ERD’ magnitude) when nociceptive somatosensory stimuli were delivered to the left hand for right-handed subjects (Figs. 1, 3, 4).

### Limitations

There are two potential limitations in the present study that needs for further investigations. First, since subjective ratings at each stimulus intensity were different for different sensory modalities, it implies that the stimuli belonging to different sensory modalities used in our experiment might not be perfectly matched. It could be a result of the asymmetrical experiment manipulation between the nociceptive and non-nociceptive sensory stimuli. Specifically, the intensities for the nociceptive somatosensory stimuli were established for each subject before the experiment, while stimulus intensities for other sensory modalities were pre-determined and fixed for all subjects. Second, both behavioral and EEG responses to sensory stimuli could be sensitive to the spatial configurations of the sources of stimuli, e.g., the distance between the source of the stimulation and the body and the distance between both hands^48–50^. However, we did not manipulate the spatial configurations of the sources of stimuli in the present study. Therefore, the negative “stimulated side” effect for the non-nociceptive sensory stimuli might be only restricted to the spatial configuration applied in the present study.

## Conclusions and implications

Altogether, our results suggested that the sensitivity to detect transient nociceptive stimuli was higher for the non-dominant hand (i.e., the left hand) than the dominant hand (i.e., the right hand) for right-handed subjects. This laterality phenomenon would be important to provide a prompt reaction to noxious events, which may help compensate for the worse motor performance of the non-dominant hand^17,18^. Theoretically, the laterality phenomenon would be contributed by both peripheral (use-dependent changes of the thermal and mechanical properties of the skin and the underlying tissues of the dominant hand) and central (the functional asymmetry in the cerebral organization) factors. Practically, the laterality phenomenon should be considered when designing experiments for pain laboratory studies and evaluating regional sensory abnormalities for patients with clinical pain.

## Methods

### Participants

A total of 100 healthy right-handed volunteers were recruited in the present study (63 females; mean age 21.57 ± 1.74 years, range from 18 to 26 years). All participants had no history of chronic pain, major medical or psychiatric illness, and no alcohol or drug abuse. They reported normal audition and normal or corrected-to-normal vision.

### Ethics

The study was approved by the ethical standards of the Institutional Review Board of the Institute of Psychology, Chinese Academy of Sciences. All experiments were performed in accordance with all relevant guidelines and regulations. The informed consent forms were signed by all participants before the experiment.

### Sensory stimulation

**Figure 5:**
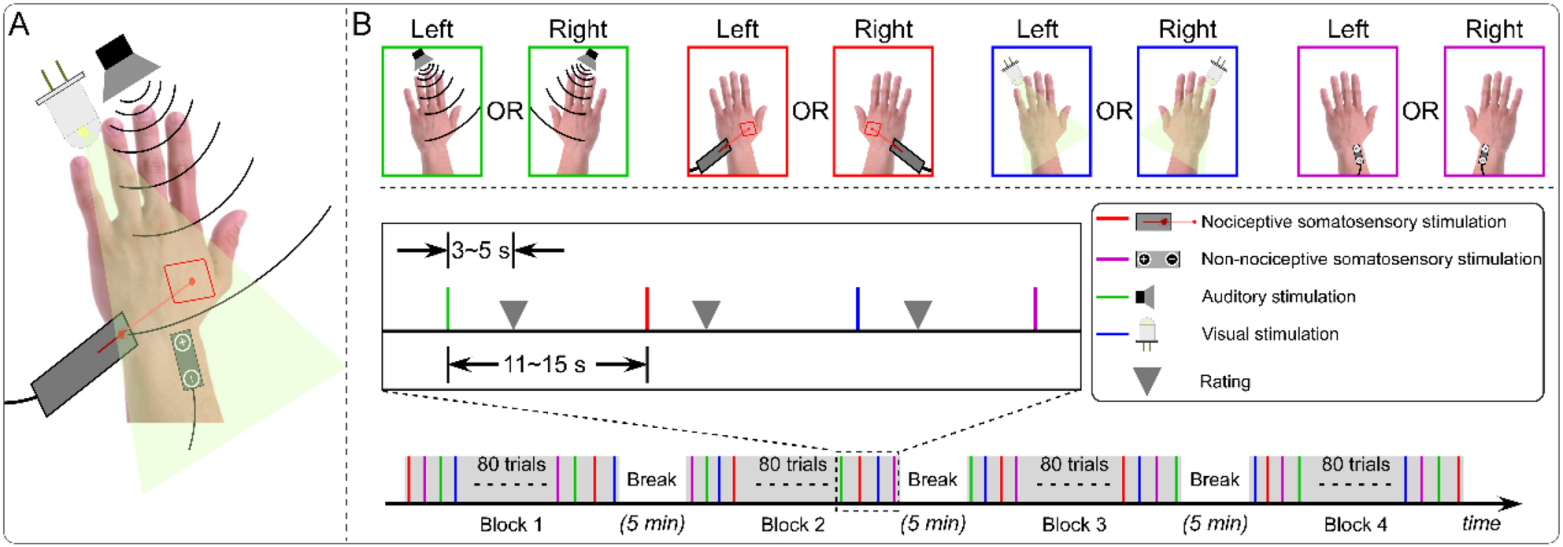
Experimental design. (**A**) Transient stimuli belonging to four different sensory modalities (nociceptive somatosensory, non-nociceptive somatosensory, auditory, and visual). (**B**) The experiment consisted of four blocks, and in each block, there were 80 trials with transient stimuli belonging to four different sensory modalities (nociceptive somatosensory, non-nociceptive somatosensory, auditory, and visual). In two blocks, sensory stimuli were delivered to subject’s left side, and in the other two blocks, sensory stimuli were delivered to subject’s right side. The order of sensory modalities was pseudo-randomized, and the order of stimulated sides was counterbalanced across subjects. After each sensory stimulus, an auditory tone was delivered randomly between 3 and 5 s to prompt subjects to rate the perceived intensity on the 0–10 NRS verbally. The inter-stimulus interval (ISI) varied randomly between 11 and 15 s, and the interval between two consecutive blocks was 5 min.

Subjects were presented with transient stimuli belonging to four different sensory modalities: nociceptive somatosensory, non-nociceptive somatosensory, auditory, and visual (Fig. 5A). Nociceptive somatosensory stimuli (i.e., nociceptive-specific radiant-heat stimuli) were generated by an infrared neodymium yttrium aluminum perovskite (Nd: YAP) laser with a wavelength of 1.34 μm and a pulse duration of 4 ms (Electronical Engineering, Italy)^51^. A He–Ne laser pointed to the area to be stimulated, and the laser beam with a diameter of approximately 7 mm was transmitted via an optic fiber. Laser pulses were delivered to a pre-defined squared area (5 × 5 cm^2^) on the dorsum of subject’s left or right hand. After each stimulus, the beam target was moved by ∼ 1 cm in a random direction to avoid nociceptor fatigue or sensitization. Subjects were required to rate the intensity of pain perception on a 0–10 numerical rating scale (NRS) after each stimulus, with 0 standing for “no sensation” and 10 standing for “the strongest sensation imaginable”. Since pain perception varies widely among different subjects (i.e., the same laser stimulus can elicit unbearable painful sensation in one subject, but be barely perceived by another subject)^26^, the laser energies were individually determined using the ascending method of limits: for each subject, the four laser energies that evoked subjective ratings of ~ 2, 4, 6, and 8 on the 0–10 NRS by increasing the stimulus energy in steps of 0.25 J until the target rating was obtained. Such procedure repeated three times for each hand, and laser energies averaged across tests and hands were used for the formal experiment. Across subjects, the stimulus energies were as follows: E1, 2 ± 0.22 J; E2, 2.7 ± 0.25 J; E3, 3.4 ± 0.31 J; and E4, 4.1 ± 0.39 J.

Non-nociceptive somatosensory stimuli were constant current square-wave electrical pulses with a duration of 1 ms (model DS7A, Digitimer, UK) delivered through a pair of surface round electrodes with a diameter of 1.6 cm and an inter-electrode distance of 1 cm. The electrodes were placed at the wrist of subject’s left or right hand. Since the delivered electrical pulses were perceivable and bearable for all subjects, the stimulus intensities used in the experiment were identical for all subjects. Specifically, stimulus intensities were determined based on a preliminary psychophysical experiment performed on 10 age- and sex-matched subjects, to ensure that subjective ratings were ~ 2, 4, 6, and 8 out of 0–10 for E1 to E4, respectively (the same procedures were performed to determine the stimulus intensities of auditory and visual stimuli). All subjects were asked to assess the intensity of the electrical stimulation they had received during the preliminary experiment, and none reported painful sensation. The stimulus intensities were as follows: E1, 2 mA; E2, 3 mA; E3, 5 mA; and E4, 7 mA.

Auditory stimuli were brief 800-Hz pure tones with a duration of 50 ms (5-ms rise and fall time) delivered by a speaker positioned in front of the subject’s left or right hand. The same as non-nociceptive somatosensory stimuli, the stimulus intensities, determined based on the preliminary experiment, were identical for all subjects (E1, 65 dB; E2, 69 dB; E3, 75.5 dB; and E4, 80 dB).

Visual stimuli were brief flashes with a duration of 5 ms delivered through a white light-emitting diode placed on top of the speaker, which was close to the subject’s left or right hand, and pointed toward the subject’s eyes. For all subjects, the stimulus intensities, also determined based on the preliminary experiment, were as follows: E1, 0.3 lx; E2, 1.3 lx; E3, 40.5 lx; and E4, 53.9 lx.

## Experimental procedure

The experiment was performed in a dim, silent, and temperature-controlled room. Subjects sat on a comfortable chair and were asked to relax their muscles and focus their attention on the stimulation. They were instructed to keep their gaze on a cross placed centrally in front of them, at a distance of ~ 1 m, 30° below eye level. The experiment consisted of four successive blocks: for two blocks, sensory stimuli were applied to subjects’ left side, while for the other two blocks, sensory stimuli were applied to their right side. For each sensory modality, stimulated side, 10 trials of each stimulus intensity were delivered, resulting in a total of 320 trials (4 sensory modalities × 2 stimulated sides × 4 stimulus intensities × 10 trials = 320 trials). The orders of sensory modalities and stimulus intensities were pseudo-randomized, and the order of stimulated sides (left and right) was counterbalanced across subjects. During the experiment, the subjects placed their both hands on the table about 60 cm apart. To minimize the possible influence of different spatial locations of the stimulation, all sensory stimuli were delivered on or near the dorsum of subjects’ left or right hand. The distance between the source of stimulations and the subjects’ heads was approximately 60 cm. To prevent the subjects from seeing the laser beam and generating expectations for the upcoming stimulus, their hands were blocked by a baffle when presented with laser stimuli. The inter-stimulus interval (ISI) varied randomly between 11 and 15 s, with a rectangular distribution. After each sensory stimulus, an auditory tone was delivered randomly between 3 and 5 s to inform subjects to rate the perceived stimulus intensity on the same 0–10 NRS (Fig. 5B). To familiarize the subjects with the stimulation, a small number of stimuli with different stimulus intensities were delivered for each sensory modality before the formal experiment. To ensure tactile rather than painful sensation evoked by the electrical stimulation, all subjects were asked to assess the intensity of the electrical stimulation they had perceived during this familiarization phase, and none reported painful sensation. To rule out the possible influence of skin temperature on perceived pain perception, the surface temperature of the dorsum of both hands was measured for each subject before each block using an infrared thermometer^52,53^. The temperature was 33.21 ± 1.1 °C and 33.20 ± 1.1 °C for left and right hands respectively (*t*(99) = 0.109, *p* = 0.914, paired-sample t-test).

### EEG data recording

EEG data were acquired via 64 Ag–AgCl scalp electrodes placed according to the international 10–20 system (Brain Products GmbH, Germany). The nose was used as the reference, and electrode impedances were kept below 10 kΩ. Signals were digitized using a sampling rate of 1000 Hz and a band-pass filter from 0.01 to 100 Hz. To monitor ocular movements and eye blinks, electrooculographic (EOG) signals were simultaneously recorded using two surface electrodes, one placed over the lower eyelid and the other placed ~ 1 cm lateral to the outer corner of the orbit.

### EEG data preprocessing

EEG data were pre-processed using EEGLAB^54^. Continuous EEG data were band-pass filtered between 1 and 100 Hz, and segmented into 3-s epochs (− 1 to 2 s relative to stimulus onset). After baseline correction using the prestimulus interval, epochs contaminated by artifacts due to gross movements were removed, and signals contaminated by eye blinks and movements were corrected using an independent component analysis algorithm (runica)^54^.

### Time domain analysis

For each subject, epochs belonging to the same sensory modality (nociceptive somatosensory, non-nociceptive somatosensory, auditory, and visual) and stimulated side (left and right) were averaged, yielding eight average waveforms time-locked to the stimulus onset for each electrode. For all sensory modalities, peak-to-peak amplitudes of the vertex potentials (i.e., the N2–P2 complex) were measured from single subject average waveform for each stimulated side. Please note that we decided to measure the peak-to-peak amplitudes of the vertex potentials to minimize the influence of low-frequency drifts in EEG signals on either the N2 or P2 amplitude when the two peaks were considered separately^11,55^. The N2–P2 complex was defined as the most negative and positive deflections between 100 and 500 ms after stimulus onset at the vertex (Cz-nose)^23,56^. Please note that vertex potentials elicited by intense and transient stimuli belonging to different sensory modalities are functionally similar^10^, and the same nomenclatures (i.e., the N2 and P2) were used for all sensory modalities in the present study. In addition, for nociceptive and non-nociceptive somatosensory modalities, peak amplitudes of N1 wave were measured from single subject average waveform for each stimulated side. The N1 wave, defined as the most negative deflection preceding the N2 wave, can be optimally detected at the central electrode contralateral to the stimulated side referenced to Fz (Cc-Fz)^24,25^. Different from the vertex potentials that can be detected from all subjects, the N1 wave (i.e., a unique and clear negative deflection preceding the N2–P2 complex) was clearly identified from 80 subjects for both somatosensory modalities and both stimulated sides. It should be noted that the latency of brain responses evoked by sensory stimuli of different modalities is highly related to the conduction velocity of the sensory inputs in afferent fibers in the peripheral and central nervous systems^10^. For this reason, the comparison of latencies was not considered in the present study. Group-level waveforms were obtained by averaging single subject average waveform for each sensory modality and stimulated side. Group-level scalp topographies at the peak latency of all waves were computed by spline interpolation.

### Time–frequency decomposition

For each sensory modality and stimulated side, time–frequency decompositions (TFD) of EEG epochs were obtained using a windowed Fourier transform (WFT) with a fixed 250-ms Hanning window. The WFT yielded a complex time–frequency estimate $F(t,f)$ at each point $(t,f)$ of the time–frequency plane for each EEG epoch, extending from − 1000 to 2000 ms (in steps of 1 ms) in the time domain, and from 1 to 30 Hz (in steps of 1 Hz) in the frequency domain. The resulting spectrogram, $P\left(t,f\right)={\left|F\left(t,f\right)\right|}^{2}$, represents the signal power as a joint function of time and frequency at each time–frequency point. The spectrogram was baseline-corrected using *subtraction* approach at each frequency $f$ (reference interval: − 800 to − 200 ms relative to stimulus onset)^57^. This reference interval was chosen to avoid the adverse influence of spectral estimates biased by windowing post-stimulus activity and padding values^58,59^.

For each subject, baseline-corrected TFDs belonging to the same sensory modality (nociceptive somatosensory, non-nociceptive somatosensory, auditory, and visual) and stimulated side (left and right) were averaged, yielding eight average TFDs. According to previous publications, intense stimuli belonging to different sensory modalities can elicit large phase-locked (event-related potentials, ‘ERP’) and non-phase-locked (event-related desynchronization at alpha frequencies, ‘α-ERD’) responses^8,26,28,57^. Accordingly, two regions of interest (ROIs) were defined to extract the magnitude of time–frequency responses within the baseline-corrected TFDs for each sensory modality (ROI 1 for ‘ERP’: 100–250 ms for nociceptive somatosensory and visual stimuli, 50–200 ms for non-nociceptive somatosensory and auditory stimuli, 1–8 Hz; ROI 2 for the ‘α-ERD’: 600–1200 ms for nociceptive somatosensory and visual stimuli, 550–1150 ms for non-nociceptive somatosensory and auditory stimuli, 8–13 Hz). For each subject, magnitudes of these time–frequency responses for each ROI were measured by computing the mean of the top 20% time–frequency points displaying the highest increase (for the ‘ERP’) or decrease (for the ‘α-ERD’) for each sensory modality and stimulated side^26,60^. Group-level scalp topographies of the magnitude of each time–frequency response (the ‘ERP’ and ‘α-ERD’) were computed by spline interpolation.

### Statistical analysis

To test the effects of sensory modality (four levels: nociceptive somatosensory, non-nociceptive somatosensory, auditory, and visual) and stimulated side (two levels: left and right) on perceptual intensities and electrophysiological responses elicited by sensory stimuli, we performed two-way repeated-measures analyses of variance (ANOVA). The Greenhouse–Geisser correction was applied in light of observed violations of sphericity assumption^61^, and the corrected degrees of freedom were reported if the equal variance assumption was violated. When there was a significant interaction between the above factors, post hoc paired-sample t-tests were performed. To account for multiple comparisons, Bonferroni corrections were performed to correct the significance level (*p* value), when necessary.

To assess whether the laterality of perceptual intensities elicited by sensory stimuli would be associated with stimulus intensity, we split all trials into low-intensity trials (i.e., E1 and E2) and high-intensity trials (i.e., E3 and E4) for each subject and each sensory modality. Please note that the two-level split operation was adopted due to the insufficient number of trials in each stimulus intensity (i.e., 10 trials). Then, we performed three-way repeated-measures ANOVA with “sensory modality”, “stimulated side”, and “stimulus intensity” (two levels: low intensity and high intensity) as within-subject factors, and ratings of perceived intensity as the dependent variable. When there was a significant interaction between the above factors, post hoc paired-sample t-tests were performed.

All statistical analyses were carried out in SPSS 17.0 (SPSS Inc., New York, USA), and the statistical significance level was set at 0.05. The effect size was estimated by partial eta-squared (*η*_*p*_^2^).
